# Authors’ Reply to ‘Pyogenic Liver Abscess and Risk of Acute Pancreatitis’

**DOI:** 10.2188/jea.JE20150109

**Published:** 2015-07-05

**Authors:** Shih-Wei Lai, Kuan-Fu Liao, Cheng-Li Lin, Pei-Chun Chen

**Affiliations:** 1 College of Medicine, China Medical University, Taichung, Taiwan; 2 Department of Family Medicine, China Medical University Hospital, Taichung, Taiwan; 3 Graduate Institute of Integrated Medicine, China Medical University, Taichung, Taiwan; 4 Department of Internal Medicine, Taichung Tzu Chi General Hospital, Taichung, Taiwan; 5 Management Office for Health Data, China Medical University Hospital, Taichung, Taiwan; 6 Clinical Informatics & Medical Statistics Research Center, Chang Gung University, Tao-Yuan, Taiwan

Dear Dr. Shen,

We sincerely thank you for your interest in our study, which was recently published in the Journal of Epidemiology.^1^ First, we agree that, in the presence of non-proportional hazards, presenting an average hazard ratio (HR) over the entire follow-up period might not be informative because the magnitude of effect changes over time. However, a methodology report has shown that period-specific HRs (ie, HRs estimated in time-partitioned periods) may have built-in selection bias.^2^ Therefore, instead of time-partitioned estimations, we presented a series of average HRs with increasingly longer follow-up time, with findings showing that the elevated risk of acute pancreatitis associated with pyogenic liver abscess steadily decreased over time and that the association persisted for 5 years (Table 2 of our paper).

Second, we agree that the strength of interaction effects of pyogenic liver abscess and comorbidities on risk of acute pancreatitis (Table 3 of our paper) may differ among time-partitioned periods. If possible, we will analyze this effect for each time-partitioned period in further research.

We appreciate your helpful comments.
